# Rapid Automated Immunohistochemistry on Frozen Sections Enables Real‐Time Surgical Pathology Decisions

**DOI:** 10.1111/apm.70145

**Published:** 2026-02-03

**Authors:** Camilla Christine Qvist, Mie Bruun Elmbak, Julie Smith, Josefine Staldgaard, Gry Lipczak, Majbritt Wagner‐Eckert, Tina Klitmøller Agander

**Affiliations:** ^1^ Department of Pathology Rigshospitalet, The Capital Region of Denmark Copenhagen Denmark; ^2^ Department of Technology, Faculty of Health University College Copenhagen Copenhagen Denmark

**Keywords:** automation in pathology, fast‐frozen section, immunohistochemistry, intraoperative diagnostics, surgical pathology

## Abstract

Intraoperative frozen section (FS) analysis is critical in surgical pathology, but conventional hematoxylin and eosin (H&E) staining has limitations in poorly differentiated neoplasms and resection margins. Immunohistochemistry (IHC) provides higher diagnostic specificity, yet has traditionally been too time‐consuming for intraoperative urgency. This study aimed to optimize, validate, and evaluate Fast Frozen Rapid Automated Immunohistochemistry (FFRA‐IHC) using the Q‐Stain X Autostainer to improve surgical diagnostics. After optimization with antibodies CKAE, CK5, CK7, CD45, and Synaptophysin, 44 tissue samples from patients undergoing surgery at Rigshospitalet, Copenhagen, were analyzed by FS H&E, FFRA‐IHC, and standard formalin‐fixed paraffin‐embedded (FFPE) methods. Automated FFRA‐IHC showed high diagnostic accuracy and supported immediate clinical decision‐making: of 37 tumors not classifiable by FS H&E alone, 25 (68%) were classified into specific tumor types, and all five ambiguous resection margins were resolved. A cost–benefit analysis indicated efficiency gains with reduced hands‐on time compared to manual IHC. In conclusion, FFRA‐IHC demonstrated promising results for enhancing intraoperative diagnostics, leading to its implementation in the daily workflow at our department. Future studies should expand antibody panels and assess the broader clinical impact to further improve intra‐ and perioperative care.

## 1 Introduction

Frozen section (FS) diagnosis using rapid hematoxylin and eosin (H&E) staining is a well‐established method to guide surgical interventions by evaluating resection margins and characterizing indeterminate lesions in terms of histological type and origin. FS H&E meets the urgent demands of intraoperative diagnostics due to its rapid turnaround time [1, 2]. Although generally sufficient, FS H&E may be inadequate in cases involving poorly differentiated tumors or ambiguous resection margins, where H&E alone cannot reliably provide a definitive histopathological diagnosis [3, 4]. Under such circumstances, enhanced diagnostic sensitivity and specificity are essential to guide surgical decision‐making and potentially reduce the need for subsequent reoperative interventions [3, 4].

Conventional immunohistochemistry (IHC) on formalin‐fixed paraffin‐embedded (FFPE) tissue can enhance diagnostic precision; however, it requires multiple preparatory steps and automated staining protocols with turnaround times of two to 4 h [5]. This extended processing time limits the applicability of conventional IHC in intraoperative FS diagnostics. Manual IHC on frozen sections is labor‐intensive, time‐consuming, and characterized by low reproducibility [4, 6], reinforcing the reliance on H&E staining in urgent surgical contexts despite its limitations [7].

The integration of fast frozen rapid automated immunohistochemistry (FFRA‐IHC) holds significant promise for improving diagnostic accuracy. FFRA‐IHC has the potential to increase sensitivity when assessing ambiguous resection margins and to enhance specificity in evaluating tumors of unknown type and origin [3]. By providing rapid and reliable diagnostic information, FFRA‐IHC can reduce intraoperative uncertainty, support more accurate surgical decision‐making, and decrease the likelihood of reoperations. In addition, FFRA‐IHC can substantially shorten the time from tissue sampling to diagnostic reporting, reducing the waiting period from several days to mere hours. This acceleration may improve patient outcomes, adherence to treatment, and satisfaction by enabling timely clinical decision‐making [8, 9, 10]. Evidence demonstrates a direct correlation between patient satisfaction and the duration of waiting time for investigation results, and early access to pathology reports may contribute to reducing health inequalities, as observed in optimized diagnostic workflows for Ear‐Nose‐Throat (ENT) cancer patients at Rigshospitalet [10].

Although FFRA‐IHC methodologies have been described in the literature, existing approaches are often limited in scope, scalability, or efficiency, with some remaining time‐intensive [6, 11, 12, 13, 14]. Consequently, a fully automated, rapid, and reproducible platform suitable for intraoperative diagnostics is still required.

At our Department of Pathology at Rigshospitalet, over 8500 FS analyses are conducted annually, covering both margin evaluations and tumor classification. The department's quality standard requires a maximum turnaround time of 20 min from tissue receipt to pathologist report. This capability aligns with institutional quality criteria and has been validated in other laboratory settings [2, 15, 16, 17].

This study aimed to optimize and validate Q‐Stain X Autostainer for FFRA‐IHC on frozen tissue sections in the intraoperative FS laboratory. A minimal antibody panel—including CK‐AE, CK5, CK7, CD45, and Synaptophysin—was selected to distinguish carcinomas, lymphomas, melanomas, and neuroendocrine tumors, as MART‐1 was unavailable at the time of study. This panel is particularly useful in cases where conventional FS H&E is inconclusive, such as tumors with ambiguous morphology or challenging resection margins. The study evaluates both turnaround time and diagnostic accuracy in real time, highlighting the potential of FFRA‐IHC to complement and enhance standard FS diagnostics while maintaining feasibility within routine clinical workflows.

## 2 Materials and Methods

### 2.1 Study Design and Patients

**FIGURE 1:**
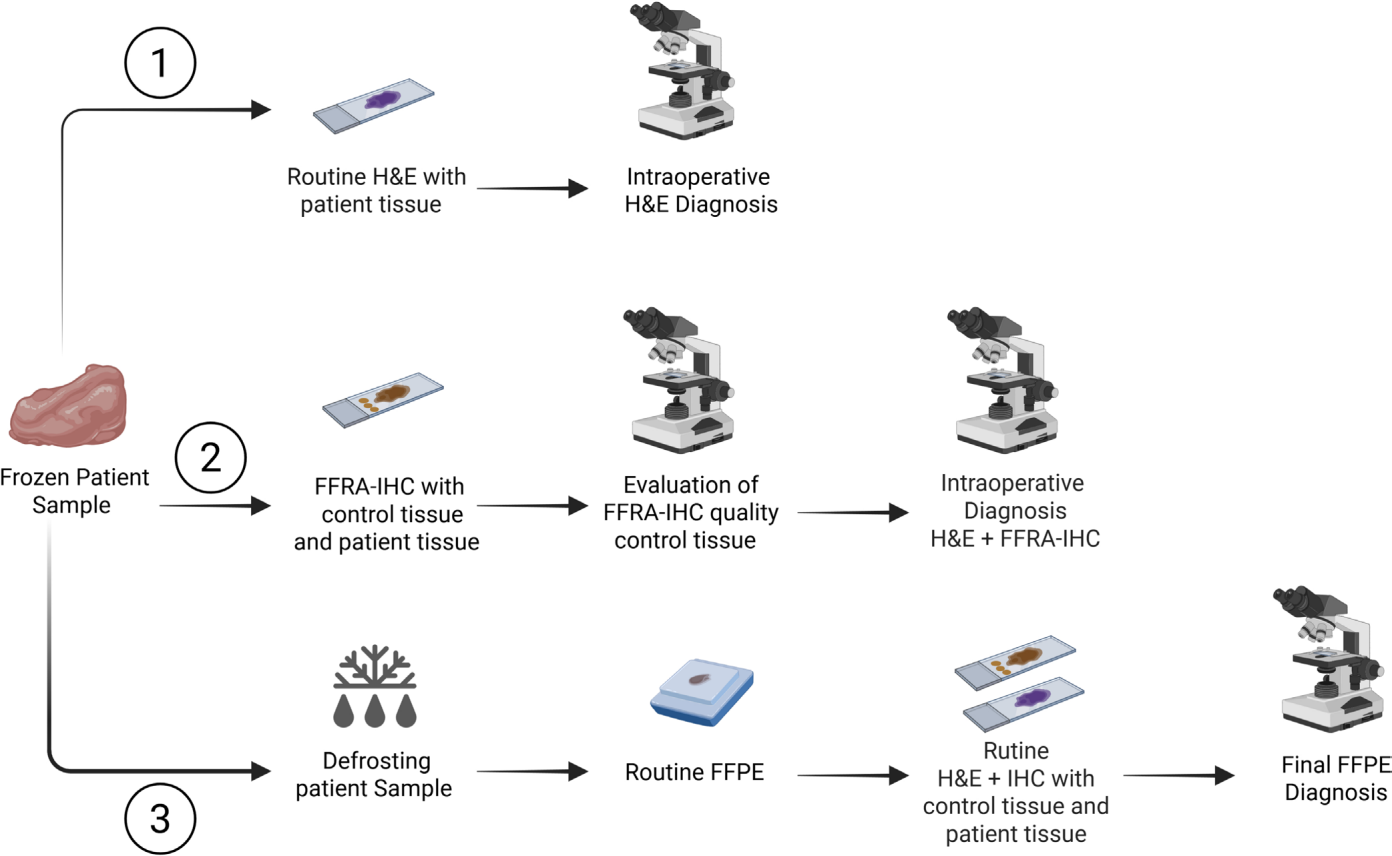
Study design. Steps 1 and 3: Conventional frozen section procedure; Step 2: Fast frozen rapid automated immunohistochemistry (FFRA‐IHC), validated in this study.

From patients undergoing surgery, tissue samples from all organ systems were submitted for conventional FS H&E analysis at the Department of Pathology, Rigshospitalet, Copenhagen, Denmark (DP RH) between September 2023 and May 2024. During this period, approximately 6300 frozen section (FS) analyses were performed. For our validation, 44 samples were identified as suitable for additional FFRA‐IHC analysisfollowing FS H&E assessment. Samples were included in the study if the pathologists determined that FFRA‐IHC would provide relevant supplementary diagnostic information (Figure 1). The recommended number of samples for validating a new antibody clone and platform generally ranges from 20 to 40 tissue specimens [18], and the institutional guideline recommendation is 30 samples [19]. The study resulted in 44 samples for FFRA‐IHC from 44 patients, comprising 13 benign and 31 malignant tumors and 5 resection margins across various organ systems. The mean age of patients was 58, ranging from 26 to 89 years, with 26 men and 18 women. All samples underwent verification and were registered in the Danish Tissue Utilization Register. The Research Ethics Committee at Rigshospitalet determined that no further ethical approval was necessary. The study was conducted in accordance with guidelines from the Danish Data Protection Agency regulations to ensure patient consent and anonymity.

### 2.2 Routine Frozen Section Procedure

All samples for FS evaluation were cryopreserved and embedded in Cryo Embedding Compound (MCC) at −40°C using PrestoCHILL (Milestone, Italy). The tissue block was fast frozen at −40°C and sectioned at 5 μm in −20°C on a Cryostar NX70.

The sectioned slides were briefly fixed in 70% ethanol before staining. The FS H&E staining protocol proceeded as follows: slides were immersed in hematoxylin for 2 min, rinsed in water, dipped 10 times in sodium carbonate, rinsed again in water, dipped 20 times in eosin, quickly rinsed in water, dehydrated in 99% ethanol, and mounted with tissue mount. Following staining, the sections were promptly examined by a pathologist as a part of the routine setting.

### 2.3 Control Tissue

Control tissue was included on all FFRA‐IHC slides to ensure staining quality and reproducibility. Control material was derived from a multiblock containing unfixed normal colon, tonsil, skin, and liver tissue, prepared according to NordiQC guidelines [20]. Biopsies of 4 mm were cryopreserved at −80°C and sectioned at 5 μm using a Cryostar NX70 (Thermo Fisher Scientific, USA) before being mounted onto FLEX IHC slides (Agilent, Denmark). Each control slide was stored at −20°C until use.

### 2.4 FFRA‐IHC Procedure

For FFRA‐IHC, tissue sections were thawed to room temperature and fixed in acetone for 3 min. Slides were transferred to wash buffer to prevent drying, then loaded into the Q‐Stain X Autostainer. FFRA‐IHC was performed following the optimized protocol presented in Table 1, which was applied for all antibodies included in the panel (CK‐AE, CK5, CK7, CD45, and Synaptophysin). Reagents were used according to the manufacturer's instructions, except that the blocking reagent was replaced with an endogenous peroxidase blocker (DAKO) to improve staining specificity. Automated staining was conducted at 30°C, followed by dehydration in 99% ethanol and mounting. Each slide chamber could be reused up to five times, with cleaning between runs to prevent cross‐contamination.

**TABLE 1:** Protocol for Q‐Stain X. All automated steps were performed at 30°C set by the instrument.

Protocol for Q‐Stain X	Time
Manually	
Fixation in acetone	3 min
Place in washbuffer	—
Transfer slide to slidechamber	—
Automated	
Wash with washbuffer	—
Blocking for endogen peroxidase	1 min
Wash with washbuffer	—
pHRP‐Ab	3 min
Wash with washbuffer	—
Enhancer	3 min
Wash with washbuffer	—
DAB + substrate	2 min and 30 s
Wash with washbuffer	—
Wash with DI water	—
Hematoxylin counterstain	5 s
Manually	
Dehydration 99% ethanol	—
Mounting with tissue mount	—

*Note:* All the incubation times for the automatic steps are according to the platform's settings. pHRP‐Ab = poly horse radish peroxidase antibody. Reagents: Blocking (endogen peroxidase) (Dako, Denmark). pHRP‐Ab, enhancer and DAB+substrate (Novodiax, USA).

Abbreviation: DAB, diaminobenzidine.

### 2.5 Reagents

Reagents specific to the Q‐Stain X system and developed by Novodiax were used in this study (Table 1). All reagents were stored and handled according to the manufacturer's guidelines and were ready‐to‐use (RTU) without further dilution. The antibody panel included CK‐AE, CK5, CK7, CD45, and Synaptophysin. Based on our optimization, the Novodiax blocking reagent was substituted with an endogenous peroxidase blocker from DAKO (Denmark), also in RTU form, and transferred to a Q‐Stain X‐compatible cartridge.

### 2.6 Diagnostic Impact of FFRA‐IHC

Initially, the pathologist rendered a preliminary diagnosis based on FS H&E staining. This was followed by an FFRA‐IHC analysis to refine the diagnosis and assess discrepancies or changes. The diagnostic result was conveyed in real time to the surgeon during surgery. After these assessments, the tissue was thawed, formalin‐fixed, and paraffin‐embedded (FFPE), sectioned, and stained—the gold standard for definitive diagnosis. In this study, the initial FS H&E and FFRA‐IHC diagnoses were compared to the final FFPE‐based diagnosis, providing a comprehensive evaluation of diagnostic alignment (see study design in Figure 1).

**FIGURE 2:**
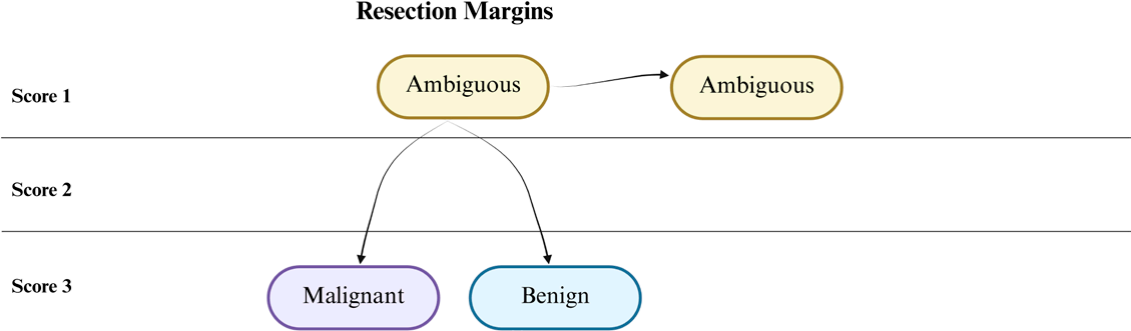
Scoring methodology for resection margins. Score 1: FFRA‐IHC does not change FS H&E diagnosis (still ambiguous). Score 3: FFRA‐IHC results in a definitive diagnosis (benign or malignant). Scores were confirmed by comparison with the final FFPE‐IHC diagnosis.

To assess the impact of FFRA‐IHC, two semi‐quantitative scoring systems were prepared: one for resection margins and one for tumor diagnostics. Scores were assigned after the final FFPE‐IHC diagnosis. For resection margins, FS H&E distinguished three outcomes: non‐malignant, malignant, or “await final diagnosis” when tumor involvement was ambiguous. In the latter case, FFRA‐IHC resolved the uncertainty, scoring +3 points if the result was comparable to the final diagnosis (Figure 2).

**FIGURE 3:**
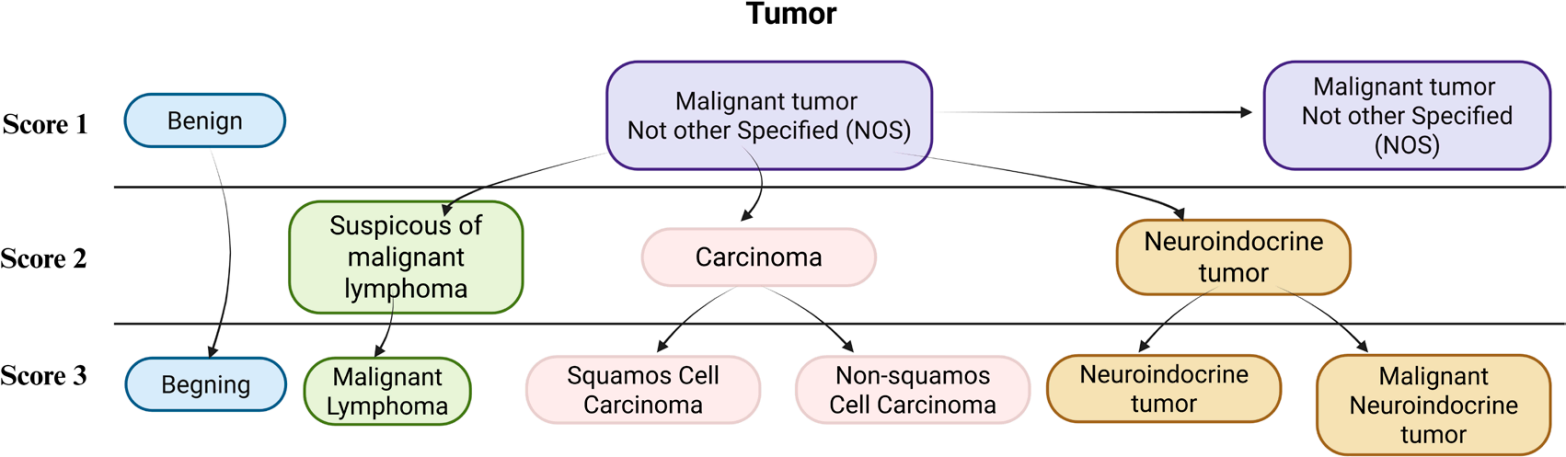
Scoring methodology for tumor classification and concordance. FFRA‐IHC allows reclassification of malignant tumors labeled as Not Otherwise Specified (NOS) into distinct categories: Carcinoma (CK‐AE+), suspected lymphoma (CD45+), neuroendocrine tumor (Synaptophysin+), squamous cell carcinoma (CK‐AE+/CK5+/CK7−) and non‐squamous carcinoma (CK‐AE+, CK5‐, CK7+/−). Benign tumors are included as Score 1. Scores 2–3 reflect increasing diagnostic specificity. All categorizations were confirmed by comparison with the final FFPE‐IHC diagnosis.

For tumor diagnostics, when FS H&E yielded “Malignant tumor Not Otherwise Specified (NOS), awaiting further analysis and final diagnosis,” the impact of FFRA‐IHC was evaluated as an additional analysis (Figure 3). If the pathologist broadly categorized tumors from FFRA‐IHC as carcinoma (CK‐AE+) or suspicious of lymphoma (CD45+), this corresponded to +2 points in the scoring system. More specific categorization based on FFRA‐IHC—such as malignant lymphoma (CD45+), neuroendocrine tumor (Synaptophysin+), or distinguishing squamous cell carcinoma (SCC; CK‐AE+/CK5+/CK7−) from non‐SCC (CK‐AE+, CK5−, CK7+/−)—corresponded to +3 points. Benign tumors were also included to evaluate overall diagnostic efficiency (see Section 2.9).

### 2.7 Evaluation of FFRA‐IHC Controls

The control tissue on each FFRA‐IHC slide was evaluated after diagnostics by two independent biomedical laboratory scientists (BLS) for each antibody. Positive and negative reactions in the control tissues were reported including if the reaction was nuclear, membranous, or cytoplasmatic. This was compared to the standard positive reaction of the antibody, which is described in Table 2. The criteria for exclusion were no positive staining at all or overstaining, including high background staining.

**TABLE 2:** Characteristics of staining pattern for five antibodies: CK‐AE, CK5, CK7, CD45, and Synaptophysin.

Antibody	Staining description	Antibody type	Species reactivity	Clone	Concentration	Company
Pan Cytokeratin (CK) AE1/AE3 Abs	Cytoplasmic staining in epithelium; dot‐like pattern in certain tumors (neuroendocrine neoplasms).	Monoclonal	Mouse anti‐human	AE1/AE3	Ready to use	Novodiax, USA
Cytokeratin 5 (CK5) Ab	Diffuse cytoplasmic staining in epidermis and various epithelial cells; perinuclear enhancement.	Monoclonal	Mouse anti‐human	R226	Ready to use	Novodiax, USA
Cytokeratin 7 (CK7) Ab	Membranous/cytoplasmic staining, varying from weak to strong in many epithelia and tumors.	Monoclonal	Mouse anti‐human	R284	Ready to use	Novodiax, USA
Cluster of Differentiation 45 (CD45) Ab	Membranous/cytoplasmic staining in white blood cells (granulocytes, lymphocytes, eosinophils, etc.).	Monoclonal	Mouse anti‐human	C95	Ready to use	Novodiax, USA
Synaptophysin Ab	Diffuse cytoplasmic staining; marker of neuroendocrine differentiation.	Monoclonal	Mouse anti‐human	R1003	Ready to use	Novodiax, USA

### 2.8 Comparison of Operational Parameters in IHC Methods: Manual FS IHC Versus FFRA‐IHC

In a parallel pilot study, we tested the difference in cost–benefit between manual FS IHC and FFRA‐IHC methods through constructive technology assessment (CTA) [21]. A radar chart was generated based on multivariate data for both methods across five categories: turnaround time (20‐min limit), hands‐on time (resource efficiency), ease of training and operational efficiency (usability), personnel exposure to hazardous chemicals (safety considerations), and cost per slide (economic feasibility). Tests for each category were conducted during FFRA‐IHC on our 44 samples from this study and observed by two evaluators. Each category was given a score from 1 to 5. Definitions and elaboration of each score are shown in Table 3. For manual FS IHC the “rapid EnVision procedure” was used from Kämmerer et al. [22, 23].

**TABLE 3:** Elaboration of the five categories included in constructive technology assessment (CTA) used to compare Manual RIHC and FFRA‐IHC; turnaround time in minutes (min.), hands‐on time in % (from fixation to mounting), usability (Score 0–5, where 5 indicates the highest ease of use), exposure to hazardous chemicals and cost per slide in euro (EUR). Definition of each score from 1 to 5.

Category	Elaboration	Definition of each score				
		1	2	3	4	5
Turnaround time	Time passed from fixation of tissue to mounting with coverslip.	40–50 min	30–40 min	20–30 min	10–20 min	0–10 min
Hands‐on time^a^	Time actively spent on the analysis.	80%–100%	60%–80%	40%–60%	20%–40%	0%–20%
Usability		0–1/5	2/5	3/5	4/5	5/5
Exposure to hazardous chemicals^b^	Chromogen; 3,3′‐diaminobenzidine	Long exposure	Moderate exposure	Fair exposure	Minimal exposure	No exposure
Cost per slide	The total cost per slide includes all reagents used for the analysis.	~80–100	~60–80	~40–60	~20–40	~0–20

^a^ Lower percentage indicates less time actively spent on the analysis.

^b^ Long exposure indicates extended or frequent contact with the chromogen 3,3′‐diaminobenzidine (DAB) during the analysis.

### 2.9 Statistical Methods

Sensitivity, specificity, and concordance with FFPE IHC were calculated to evaluate the validity of the FFRA‐IHC approach. Diagnostic sensitivity and specificity were assessed using FFPE results as the reference “gold standard.” Since tumor‐free samples were not included, benign tumors were classified as “true negative” and malignant tumors as “true positive” to test the reliability of the diagnostic approach. Overall staining efficiency was determined by the proportion of successfully stained slides relative to the total number of slides processed.

## 3 Results

### 3.1 Evaluation of FFRA‐IHC Controls

A total of 44 samples resulted in 90 FFRA‐IHC slides, with varying representation of antibodies: CK‐AE (*n* = 29), CK5 (*n* = 22), CK7 (*n* = 17), CD45 (*n* = 14), and Synaptophysin (*n* = 8). One slide was excluded due to negative reactions in control tissue, and another patient was excluded due to unsuccessful FFRA‐IHC caused by missed counterstain with hematoxylin. Consequently, 42 samples and 83 FFRA‐IHC slides were included in the final dataset: CK‐AE (*n* = 27), CK5 (*n* = 20), CK7 (*n* = 15), CD45 (*n* = 13), and Synaptophysin (*n* = 8), resulting in an overall FFRA‐IHC protocol efficiency of 92%.

**FIGURE 4:**
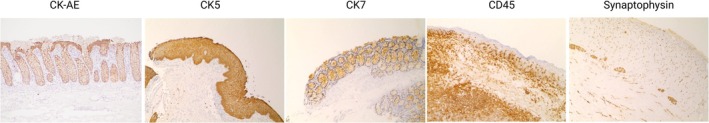
Representation Images of optimal stains for five different antibodies. Epithelial cells of the colon stained with CK‐AE. Squamous epithelial cells of the tonsil stained with CK5, while superficial epithelial cells were positive for CK7. Lymphocytes and histiocytes of the tonsil stained with CD45. Ganglion cells, axons, and neuroendocrine cells of the nerve plexus of the colon stained with Synaptophysin. All images are 100× magnification.

Optimal stains for all five antibodies are shown in Figure 4.

### 3.2 Diagnostic Impact

A total of 42 patients were scored according to our two semi‐quantitative scoring systems, including five samples with unresolved resection margins and 37 samples with unresolved tumors on FS H&E. The results are presented in Table 4a. Automated FFRA‐IHC analysis provided definitive diagnoses in all five ambiguous margins, demonstrating 100% diagnostic sensitivity and specificity for resection margin evaluation. In all cases of ambiguous margins, a panel including CD45 and cytokeratins proved the atypical cell to be lymphocytes or histiocytes and not epithelial cells.

**TABLE 4a:** FFRA‐IHC increases the diagnostic sensitivity regarding ambiguous resection margin and specifies type of tumor in unclassifiable tumors when evaluated on FS H&E alone. The numbers were small, but overall, FFRA‐IHC gave a 100% sensitivity for resection margin evaluations and 100% sensitivity for tumor diagnostics with a 68% increase in tumor type classifications.

Samples	(*n*)	Score 1	Score 2	Score 3	Sensitivity (%)	Specificity (%)
Total	42					
Resection margins	5				100	100
Specified diagnosis	5			5		
Ambiguous diagnosis	0					
Tumor diagnostics	37				100	100
Specified diagnosis	25	1	6	18		
Unaltered diagnosis	12	1		11		
Enabled tumor reclassification by 68%						

Among the 37 tumors that could not be classified based on the original FS, FFRA‐IHC enabled classification of 68% (25/37) into more specific tumor types. These 37 tumors included 20 carcinomas (various origins), 5 lymphomas, 2 neuroendocrine tumors, 8 benign lesions, 1 glioblastoma and 1 atypical meningioma (Table 4b). This detailed distribution highlights the spectrum of tumor types and the specific contribution of FFRA‐IHC in resolving previously unclassifiable tumors.

**TABLE 4b:** Breakdown of tumor types classified by FFRA‐IHC among previously unclassifiable tumors.

Tumor type	Number of cases
Carcinoma (various origins)	20
Benign lesion	8
Lymphoma	5
Neuroendocrine tumor	2
Atypical meningioma	1
Glioblastoma	1
Total	37

In all samples, the relevant diagnostic focus was preserved during sectioning. Despite the limited sample size, these results demonstrate clear clinical value for both clinicians and patients, as diagnoses were conveyed in real‐time to the surgeon during surgery. Overall, FFRA‐IHC achieved 100% sensitivity for resection margin evaluation and increased tumor type classification by 68% among tumors initially unclassifiable on FS H&E (Tables 4a and 4b).

### 3.3 Case Examples Illustrating Resolution of Diagnostic Ambiguity

In cases when frozen‐section H&E morphology alone is insufficient and final diagnosis pending, FFRA‐IHC provided decisive tumor classification. Two representative examples illustrate how rapid immunophenotyping directly influenced intraoperative decision‐making.

**FIGURE 5:**
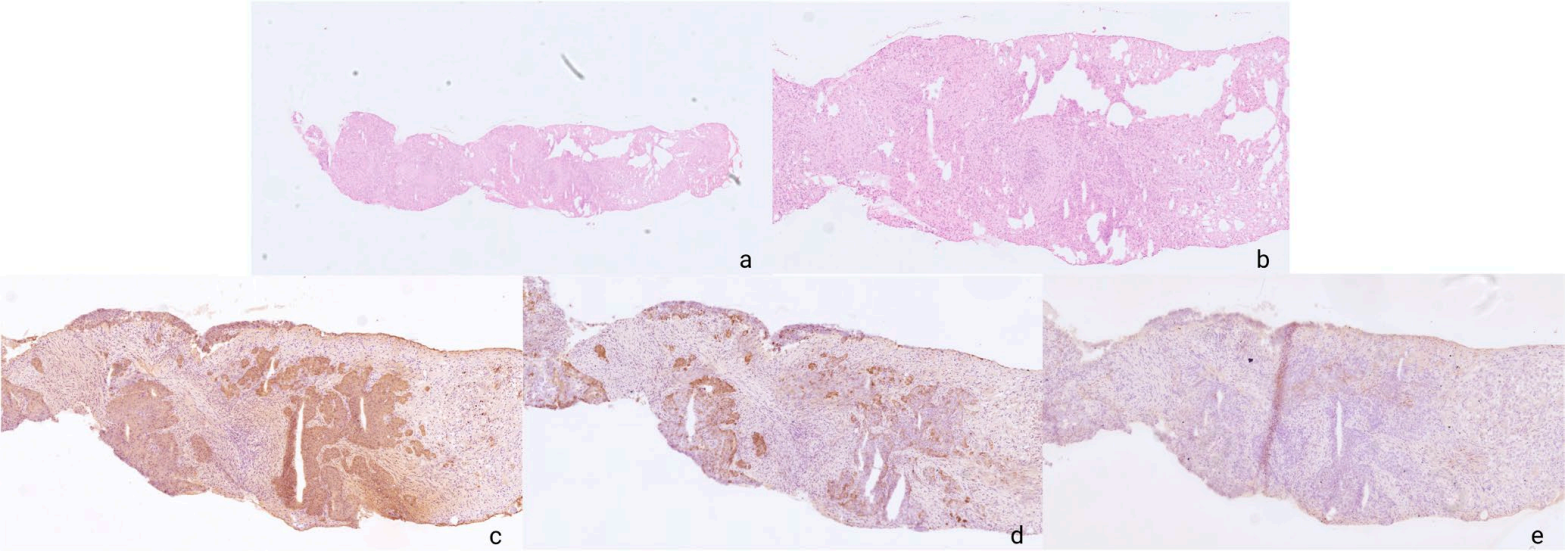
Case 1 Core needle biopsy from level 2 on the right side of the neck. Hematoxylin and eosin (FS H&E) (a ×2 magnification and b ×5 magnification) staining shows soft tissue infiltrated by malignant tumor tissue, which cannot be further classified on FS H&E sections. The frozen section diagnosis based on FS H&E staining was malignant tumor tissue; final diagnosis pending. A frozen‐section immunohistochemical analysis (FFRA‐IHC) was performed using a panel including CD45, CK5, and CK7, and was ready for microscopic evaluation after 21 min. FFRA‐IHC showed tumor cell positivity for CK5 (c ×5 magnification), focal positivity for CK7(d ×5 magnification), and negativity for CD45 (e ×5 magnification). The findings are consistent with carcinoma—most likely squamous cell carcinoma (SCC)—although it is not possible to determine whether this represents a metastasis or direct invasion. The intraoperative diagnosis enabled the surgeons to search for a potential primary tumor while the patient remained under anesthesia. The final diagnosis was metastatic SCC to level 2 with primary SCC identified in the base of the tongue.

In the first case, a poorly differentiated tumor from level 2 of the neck could not be classified on FS H&E due to a monotonous malignant cell population lacking defining morphological features. FFRA‐IHC rapidly demonstrated an epithelial immunoprofile, supporting the diagnosis of carcinoma rather than lymphoma or other non‐epithelial malignancies. This enabled the surgical team to initiate an immediate search for a likely primary site while the patient remained anesthetized, thereby altering the intraoperative workflow (Figure 5 Case 1).

**FIGURE 6:**
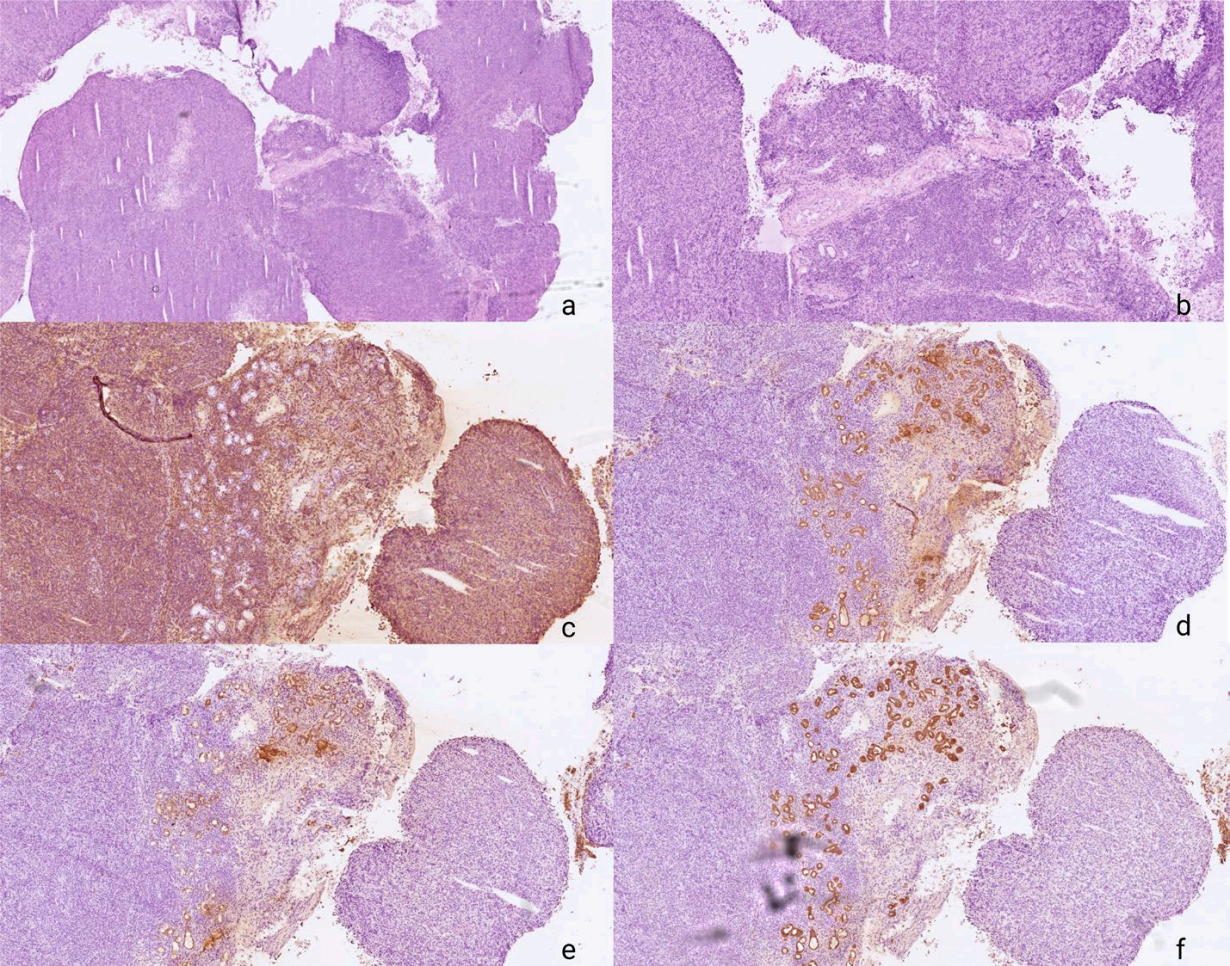
Case 2 Biopsy from the left maxillary sinus. Hematoxylin and eosin (FS H&E) staining (a ×2 magnification and b ×5 magnification) shows malignant tumor tissue that cannot be further classified. The frozen section diagnosis based on FS H&E staining was malignant tumor tissue; final diagnosis pending. A frozen‐section immunohistochemical analysis (FFRA‐IHC) including CD45, CKAE, CK5, and CK7 was completed and ready for microscopy after 23 min. The tumor cells were positive for CD45 (c ×5 magnification) and negative for CKAE (d ×5 magnification), CK5 (e ×5 magnification), and CK7 (f ×5 magnification). The findings indicate malignant lymphoma, later confirmed as diffuse large B‐cell lymphoma (DLBCL), non‐GCB phenotype. The patient was referred directly to the hematology department for urgent chemotherapy.

In the second case, a maxillary sinus lesion displayed sheets of malignant cells without clear morphological indicators of lineage. The frozen‐section diagnosis therefore remained malignant tumor unclassified on FS H&E. FFRA‐IHC performed intraoperatively showed strong leukocyte marker expression and absence of epithelial markers, shifting the interpretation from a suspected carcinoma to a high‐grade lymphoma. This rapid clarification allowed the clinical team to redirect the patient immediately toward urgent hematological evaluation rather than surgical management (Figure 6 Case 2).

Together, these cases exemplify how FFRA‐IHC can resolve diagnostic ambiguity in real time by confirming or excluding epithelial differentiation, identifying hematolymphoid origin, and guiding appropriate clinical pathways during ongoing surgical procedures.

### 3.4 Optimizations of Procedures Compared to Manufacturer's Instructions: Fixation, Slide Chamber, and Blocking Agents

To enhance staining quality and diagnostic accuracy, several procedural modifications were implemented beyond the manufacturer's guidelines after pilot testing on control tissue. Fixation time with acetone was extended from 1 min to 3 min, improving tissue stability, particularly in high‐background samples, and reducing artifact formation. This adjustment was carefully controlled to ensure fixation remained within an acceptable timeframe for rapid processing, while formalin was not tested due to potential health hazards for personnel.

The recommended Novodiax blocking reagent, based on bovine serum albumin (BSA), was substituted with an endogenous peroxidase blocker (DAKO, Denmark) to increase staining specificity. This change effectively minimized background staining, enhanced tissue contrast, and reduced non‐specific binding, supporting more precise diagnostic interpretation. Novodiax has since released a bulk‐reagent container for blocking, which may further improve efficiency; this modification was not tested in the present study.

Slide chambers presented challenges due to their small size. Slides containing both control and patient tissue sometimes received incomplete reagent coverage for larger tissue samples. Limited chamber dimensions occasionally caused air bubbles, uneven reagent distribution, and partial obscuring of tissue by the rubber membrane, resulting in false‐negative staining at tissue edges. These inconsistencies did not affect final diagnoses. In 2024, Novodiax released an updated Q‐Stain model with full‐capacity slide chambers, potentially addressing these issues; this version was not tested in this study.

Another important technical consideration was the need to prime the cartridges at instrument startup. Skipping this step resulted in incomplete reagent delivery and suboptimal staining for the first slide. Each priming process, however, generated reagent waste equivalent to one slide.

### 3.5 FFRA‐IHC Versus Manual FS IHC

**FIGURE 7:**
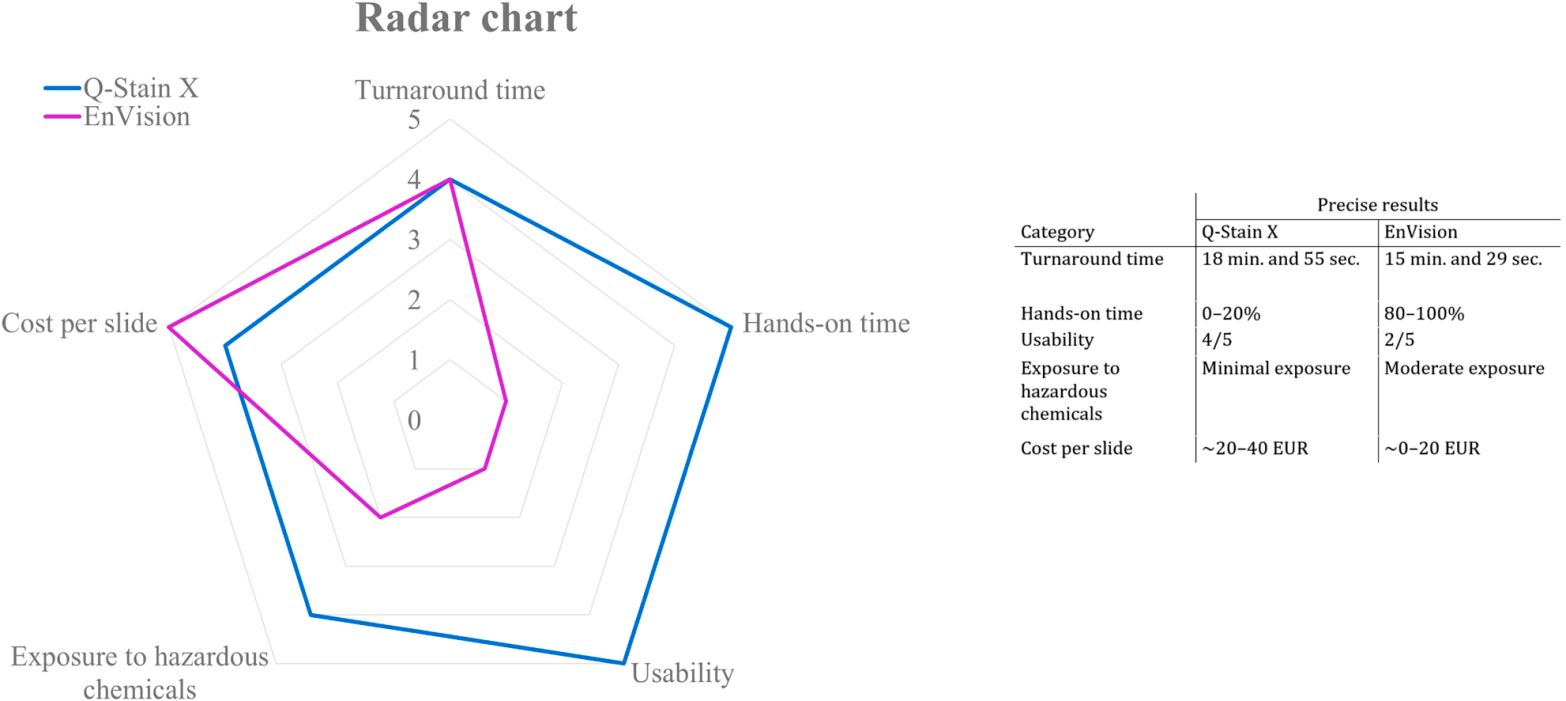
Radar chart and table displaying the scores and precise results for five categories assessed in the constructive technology assessment (CTA). The radar chart illustrates the scores (1–5) for each category, with the blue line representing Q‐Stain X (automated fast frozen rapid automated immunohistochemistry) and the purple line representing EnVision (manual frozen section immunohistochemistry). The categories include turnaround time in minutes (min.) and seconds (sec.), hands‐on time in % (time in % actively spend from fixation to mounting), usability (Score 0–5, where 5 indicates the highest ease of use), exposure to hazardous chemicals and cost per slide in euro (EUR). The accompanying table provides the precise results for each category, offering a more detailed assessment of each method's performance.

All five assessment categories were scored based on the CTA (Figure 7). FFRA‐IHC had a turnaround time of 18 min 55 s per slide with one antibody, versus 15 min 29 s for manual FS IHC, For FFRA‐IHC the turnaround time increased by ~1 min per additional slide so that the time was 19 min 55 s to analyse two slides with different antibodies. FFRA‐IHC scored higher in usability and reproducibility, while manual EnVision required more hands‐on skill, increasing the risk of human error.

Hands‐on tasks for Q‐Stain X were limited to fixation, dehydration, and slide mounting, simplifying training and enhancing reproducibility. Costs per slide were higher for the automated method, excluding instrument investment. Safety risk was reduced with Q‐Stain X, as exposure to carcinogenic DAB was limited to cartridge priming.

### 3.6 Turnaround Time

The department's quality criteria for turnaround time on frozen tissue sections is around 20 min, measured from the time the laboratory receives the specimen to the time the pathologist conveys the diagnosis to the clinicians. The average turnaround time for the FFRA‐IHC was approximately 21 min (range: 19.55–23.11 min). Consequently, the total average time from specimen receipt to final diagnosis—including tissue processing, initial H&E assessment, FFRA‐IHC, and communication of the diagnostic conclusion to the operating room—was approximately 40 min. The frozen section diagnosis based on H&E was always reported to the surgeon before FFRA‐IHC was initiated, thereby ensuring that the extended processing time remained clinically acceptable.

## 4 Discussion

This study aimed to optimize and validate a fast automated solution for analyzing FS with IHC and hence evaluate the diagnostic efficiency during real‐time intraoperative care at our hospital. Valid and reproducible staining results were achieved within a 20‐min turnaround time; hence, the platform was viable, efficient, and managed to change and improve our intra‐operative diagnostic reports to the surgeons. We also showed that hands‐on time and health hazards were reduced compared to manual IHC. Our study was limited to 42 patients, but if FFRA‐IHC continuously is rapid with valid and reproducible results, it would make a significant upgrade in the FS intraoperative laboratory, where the need for timely and accurate results is critical to surgical decision‐making, such as in evaluating resection margins and classifying tumors during surgery.

Accurate assessment of resection margins has important prognostic and economic implications, as complete tumor removal reduces recurrence risk and minimizes the need for additional surgeries or therapies [3, 4]. To overcome this, different solutions for intraoperative margin assessment have been explored like fluorescence guided surgery or microcomputed tomography, but FS still shows the best accuracy [3, 4, 24, 25]. However, manual FS IHC has been laborious and time consuming; nevertheless, FFRA‐IHC in our study demonstrated reproducibility and the potential to timely improve diagnostic sensitivity when evaluating resection margins on FS. This is valuable in surgeries like head and neck tumor resections, differentiation of breast sclerosing adenosis from infiltrating ductal carcinoma and sentinel lymph node biopsies, where quick and accurate diagnosis can directly influence surgical decision‐making and patient outcomes [3, 4]. A potential application where FFRA‐IHC would particularly benefit intraoperative fast frozen evaluation of surgical margins is during Mohs micrographic surgery of certain skin cancers [26, 27]. In a study by Sinha et al., faster solutions are sought for during Mohs surgery, and they suggest a 1‐h protocol for rapid FS immunocytochemistry [28]. We do not perform this surgery at our institution and thus this was not tested, but the FFRA‐IHC with a 20‐min time frame may be a valid alternative. Another study on FFRA‐IHC, a system called Wave (Celerus Diagnostics, USA), showed that MART‐1 could be analyzed within 16 min during Mohs surgery, which seems like better performance than in our study [13]. To our knowledge, this system is no longer available commercially, but other systems may be available. The MART‐1 antibodies were not available to the Q‐stain system but would be of great value, not only for Mohs surgery.

The FFRA‐IHC enables a more accurate pathology diagnosis to be conveyed to the surgical operating room, which can aid surgeons in making better treatment decisions during surgeries. A rapid classification of the overall tumor type can be important for the patient's further evaluation and treatment. In approximately 12% of cases (5/42), FFRA‐IHC provided information that potentially altered intraoperative management, for example distinguishing between carcinoma and lymphoma, allowing the surgeon to adjust the immediate postoperative plan. It is important to know whether the patient has disseminated carcinoma or malignant lymphoma for further evaluation. If the tumor is life‐threatening, certain tumor types are treated with radiation while others receive chemotherapy. The FS response can thus have a direct impact on the patient's acute treatment. A quick result will potentially also improve prognosis for the patient and increase patient compliance, since there is no significant delay in pathology reporting compared to conventional testing. Many patients—in particular patients with a poor social network—find it difficult to wait for the result of the diagnostic tests and complete the follow‐up program at the hospital [29, 30]. Disadvantaged groups are often lost in long‐term patient care [31]. FFRA‐IHC may contribute to balance inequality in health, because greater patient compliance may be achieved if the patient gets the plan for treatment the same day as the surgery [32].

Our findings align with studies by Brajkovic et al. [13], which highlight automated IHC's role in reducing manual workloads and improving diagnostic efficiency. However, the cost per FFRA‐IHC slide is higher than for traditional manual methods, as both the instrument and reagents cost extra. It may therefore be an analysis primarily aimed at institutions where many FS analyses are performed. In addition, clinicians are given broader opportunities for clinical decisions, such as whether the patient gets into the right investigation or treatment faster based on the more specific FS response. As for resection margins, the individual institution can review how large a proportion of the diagnoses on FS in the past year have been unresolved on FS or changed post FFPE during final microscopy. This will give the basis of whether the introduction of FFRA‐IHC would provide added value both diagnostically, clinically, and economically.

In a notable case from our study, FFRA‐IHC was pivotal in distinguishing between carcinoma and lymphoma, a diagnostic challenge that is critical due to different treatment approaches. Traditional diagnostic methods often fall short in providing definitive answers quickly, which can delay appropriate treatment. FFRA‐IHC, however, enabled the surgical team to classify the tumor rapidly, ensuring the patient received the most appropriate treatment and potentially avoiding unnecessary interventions immediately after operation.

Overall, FFRA‐IHC increased the sensitivity to 100% for resection margin evaluations and enabled a more specific tumor classification in 68% of cases. Despite our low number of patients (*n* = 5 for resections margins and *n* = 37 for tumor diagnostics), these results suggest that FFRA‐IHC may be a powerful and timely intraoperative diagnostic tool. By improving diagnostic accuracy and reducing the need for repeat surgeries, widespread adoption could have significant implications for both patient outcomes and healthcare economics.

### 4.1 Limitations and Considerations

This study was limited by its relatively small cohort (*n* = 42), single‐institution design, and restricted antibody panel. The chosen antibodies addressed a range of diagnostic needs but did not include markers such as MART‐1, essential for melanoma detection. Inclusion of such markers in future implementations could further strengthen the diagnostic power of FFRA‐IHC.

The initial investment for the Q‐Stain X system is approximately €65,709 excl. VAT, not including reagents or maintenance costs. The Q‐Stain X system's initial cost may also be a barrier, especially for smaller laboratories. Nevertheless, as the technology gains traction and production scales, costs are expected to decrease, making FFRA‐IHC more accessible to a broader range of institutions.

## 5 Conclusion

This study successfully validated the use of the Q‐Stain X Autostainer for FFRA‐IHC on FS, demonstrating that it can achieve reproducible and accurate diagnostic results within the critical 20‐min intraoperative timeframe. After this validation study, we have implented FFRA‐IHC in our daily workflow at our pathology department. FFRA‐IHC may streamline patient treatment, reduce recovery times, and lower overall healthcare costs. This is particularly impactful for underserved populations, where rapid and accurate diagnostics can make a substantial difference. In this regard, FFRA‐IHC represents not only a technological advancement but also a valuable tool for addressing healthcare disparities. Future research should explore the use of expanded antibody panels and assess the long‐term clinical impact of FFRA‐IHC in diverse surgical settings. Future studies are planned as validations with larger patient numbers to increase statistical power and generalizability.

## Conflicts of Interest

The authors declare no conflicts of interest.

## Data Availability

The data that support the findings of this study are available on request from the corresponding author. The data are not publicly available due to privacy or ethical restrictions.
